# Limited added benefit of seasonal influenza vaccination before A(H5) vaccination in mice and ferrets challenged with A(H5N1)

**DOI:** 10.1080/22221751.2026.2731495

**Published:** 2026-09-21

**Authors:** Masato Hatta, Ying Huang, Jeremy A. Duke, Joseph R. Rouse, Chenchen Feng, Xudong Lin, Li Wang, Melvyn R. Almanzar-Jordan, Zhu-Nan Li, James Stevens, Min Z. Levine, David E. Wentworth, Vivien G. Dugan, C. Todd Davis, Bin Zhou

**Affiliations:** a Influenza Division, National Center for Immunization and Respiratory Diseases, Centers for Disease Control and Prevention, Atlanta, GA, USA; b Division of Core Laboratory Services and Response, Office of Laboratory Systems and Response, Centers for Disease Control and Prevention, Atlanta, Georgia, USA

**Keywords:** A(H5) vaccine, seasonal influenza vaccine priming, pandemic preparedness, heterosubtypic immunity, clade 2.3.4.4b

## Abstract

Limited A(H5)-specific vaccine supply is expected early in a potential A(H5N1) pandemic, raising the question of whether licensed seasonal influenza vaccines could enhance protection when administered before A(H5) vaccination. We evaluated this strategy in mouse and ferret models using clade 2.3.4.4b A(H5N1) viruses. Seasonal influenza vaccination induced antibodies to seasonal haemagglutinins but did not induce detectable antibodies against A(H5) and did not consistently enhance A(H5)-directed antibody responses after A(H5) vaccination. In lethal challenge studies, seasonal vaccine priming before A(H5) vaccination was associated with improved outcomes compared with A(H5) vaccination alone in one of three mouse experiments, but this effect was not observed in the other two mouse experiments or in ferrets. These findings suggest that seasonal influenza vaccine priming provides limited added benefit to A(H5) vaccine-mediated protection against A(H5N1) under the conditions tested.

High pathogenicity avian influenza A(H5N1) viruses pose a serious pandemic threat [1–3]. Limited availability of A(H5)-specific vaccines is expected during the early months of a pandemic, and this constraint may be exacerbated by the requirement for two doses of adjuvanted A(H5) vaccines to achieve robust protection. Previous studies have established that seasonal influenza infection or vaccination can generate heterosubtypic immune responses to A(H5N1), including cross-reactive antibody and T-cell responses [4–9]. However, whether priming with a licensed seasonal influenza vaccine before A(H5) vaccination provides additional protection beyond A(H5) vaccination alone has not been specifically evaluated as a vaccination strategy. This question is distinct from whether seasonal influenza vaccination alone can confer heterosubtypic protection against A(H5N1) infection. If such a priming strategy were effective, it could support A(H5) vaccine dose-sparing approaches and expand population coverage during an A(H5N1) pandemic.

We evaluated priming with U.S.-licensed seasonal influenza vaccines (Fluzone, Flublok, or Flucelvax) [10] for its ability to enhance the protective efficacy of an experimental clade 2.3.4.4b A(H5) recombinant haemagglutinin vaccine (rH5) in animal models. Three independent mouse experiments compared adjuvanted rH5 vaccination administered with or without prior seasonal influenza vaccine priming, alongside appropriate control groups (Figure 1A–C; Supplementary Figure 1). Serological analyses using Luminex, ELISA, and haemagglutination inhibition (HI) assays demonstrated that seasonal influenza vaccines induced robust antibody responses against A(H1), A(H3), and influenza B haemagglutinins but did not induce detectable antibodies against A(H5). A single dose of rH5 elicited moderate antibody responses against A(H5) that were not significantly enhanced by prior seasonal influenza vaccination, whereas two doses of rH5 consistently induced higher A(H5) haemagglutination inhibition titres (Supplementary Table 1; Supplementary Figure 2).

**Figure 1.**
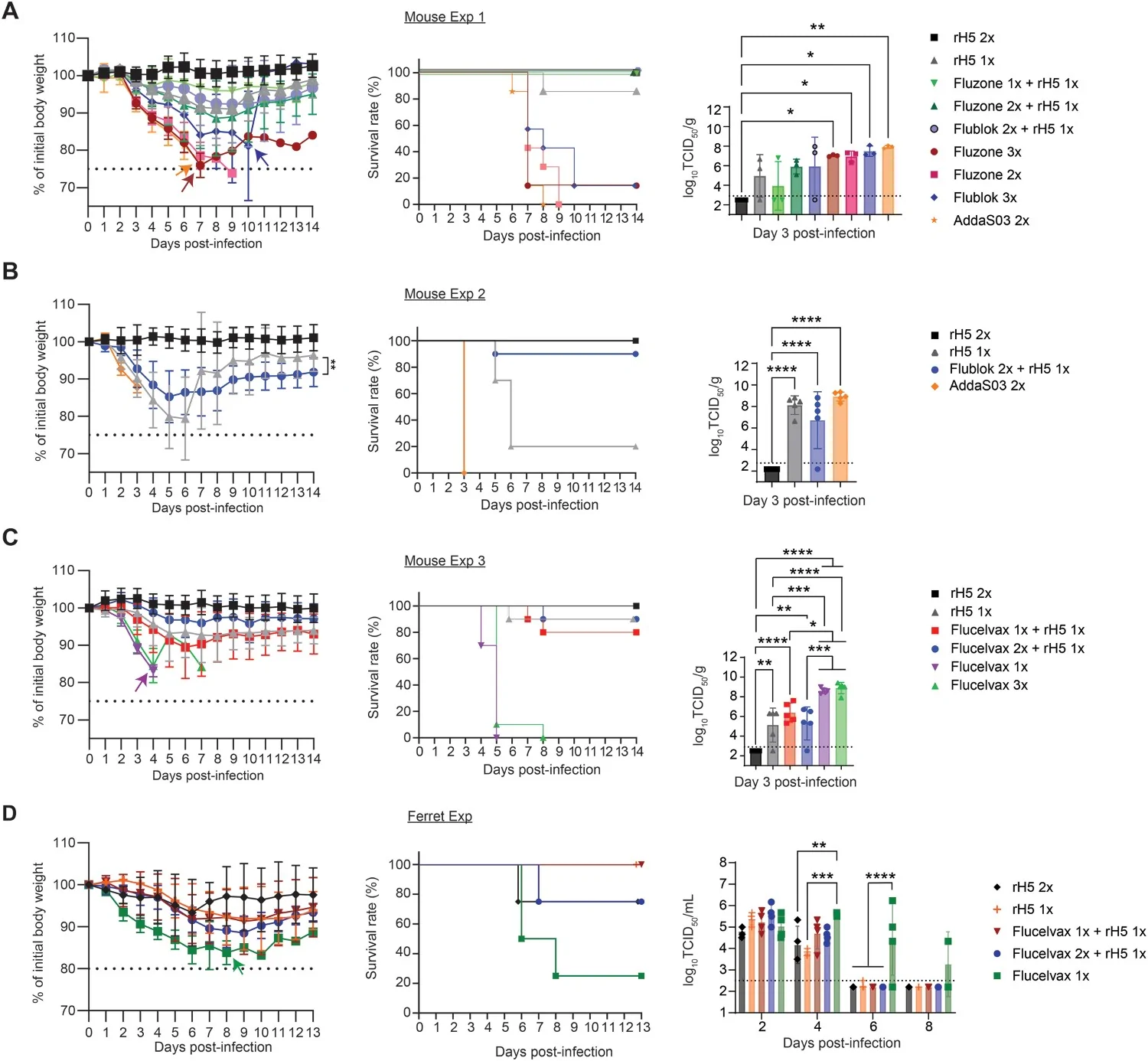
Protective efficacy of A(H5) vaccination with or without seasonal influenza vaccine priming. **(A–C)** Body weight (left), survival (middle), and lung viral titres (right) in vaccinated mice following challenge with A/Chile/25945/2023 (10^2^ TCID_50_ per mouse) in Experiment 1 (Exp 1) **(A)**; A/Texas/37/2024 (10^3^ TCID_50_ per mouse) in Experiment 2 (Exp 2) **(B)** and Experiment 3 (Exp 3) **(C)**. Mice were monitored for 14 days post-infection (dpi). Body weight (left) and survival (middle) analyses included 7 mice per group (Exp 1), 10 mice per group (Exp 2 and Exp 3), excluding animals used for organ sampling. Mice reaching humane endpoints (neurological signs, moribund behaviour, unresponsiveness, or >25% loss of baseline body weight; dotted line) were euthanised. Lung samples were collected from 3–5 mice per group on 3 dpi (right). **(D)** Ferret outcomes following challenge with A/Missouri/121/2024-like virus (10^6^ PFU per ferret; *n* = 4 per group). Body weight (left) and survival (middle) were monitored daily for 13 dpi, and nasal wash samples were collected on 2, 4, 6, and 8 dpi (right). The rH5 vaccine used in all animal experiments was based on the HA sequence of the candidate vaccine virus of A/American wigeon/South Carolina/22-000345-001/2021[13]. Where only one animal remained in a group after infection, body weight data are shown only for that remaining animal thereafter (group-matched colour arrow). Viral titres in lungs and nasal washes were determined by TCID_50_ assay. Bars indicate mean ± SD; symbols indicate individual animals. Dashed line indicates the limit of detection. Comparisons without marked significance values were not significant by Tukey’s multiple comparisons test **(right panels)**. To compare weight loss, the area under the curve (AUC) was calculated over the full post-infection monitoring period for each experiment (refer to Supplementary Material for details). Statistical comparisons indicated in the left panels represent Tukey’s multiple comparisons of AUC values between the rH5 1x group and rH5 1x groups with prior seasonal vaccination. *p < 0.05; **p < 0.01; ***p < 0.001; ****p < 0.0001.

Vaccinated mice were challenged with lethal doses of clade 2.3.4.4b A(H5N1) human isolates A/Chile/25945/2023 or A/Texas/37/2024 (Figure 1A–C). Seasonal influenza vaccination alone provided marginal protection against lethal challenge, which was more evident in mice vaccinated three times. A single dose of rH5 conferred partial protection, whereas two doses of rH5 provided complete protection as measured by survival, body weight loss, and lung viral titres. Seasonal influenza vaccine priming before rH5 vaccination resulted in moderate improvement in outcomes in one of the three mouse experiments (Figure 1B), but did not show a clear added benefit in the other two experiments (Figure 1A and C).

We conducted a similar study in a ferret model using a clade 2.3.4.4b challenge virus (A/Missouri/121/2024-like virus, MO121) that is antigenically different from the rH5 vaccine strain (Supplementary Figure 3) [11]. Seasonal influenza vaccination alone provided partial protection against a 10^6^ PFU challenge with MO121, which resulted in 100% mortality in naïve ferrets even at 10^2^ TCID_50_ (Figure 1D; Supplementary Figure 4). However, ferrets that received seasonal influenza vaccine priming followed by rH5 vaccination showed clinical outcomes and viral titres comparable to those in ferrets that received rH5 vaccination alone (Figure 1D; Supplementary Table 2 and Supplementary Figures 5). This lack of enhanced protection is consistent with serological data showing that seasonal vaccination did not induce detectable HI activity against A(H5), nor did it boost the HI titres elicited by subsequent rH5 vaccination (Supplementary Figure 6).

Prior studies have shown that seasonal influenza virus infection or vaccination can elicit cross-reactive antibody and T-cell responses to A(H5N1) antigens [4–9]. Consistent with these observations, we detected cross-reactive CD4^+^ T-cell responses following A(H1) priming, with human CD4^+^ T cells producing IFNγ upon A(H5) antigen stimulation, and observed cross-reactive memory B-cell activation in a subset of donors (Supplementary Figure 7). Similarly, CD4^+^ T cells isolated from A(H1)-vaccinated mice produced IFNγ in response to A(H5) antigen stimulation (Supplementary Figure 8). Although not measured in this study, cross-reactive N1-directed antibodies induced by seasonal influenza infection or vaccination have also been associated with reduced disease severity in A(H5N1) infection [7–9]. Additionally, pre-existing A(H3N2) immunity has been shown to confer partial protection against A(H5N1) challenge in ferrets, highlighting the complexity of heterosubtypic immunity and the potential contributions of both humoral and cell-mediated responses targeting multiple conserved viral antigens [12].

Seasonal influenza vaccination alone produced weak but detectable heterosubtypic protection against A(H5N1) challenge in both mouse and ferret models (Figure 1), including partial protection in ferrets. Thus, heterosubtypic effects remained detectable even under the stringent challenge conditions used here, although seasonal vaccination alone did not prevent mortality under these conditions. A lower challenge dose might have revealed greater protection from seasonal vaccination alone. Importantly, however, the primary objective of this study was not to assess protection conferred by seasonal influenza vaccination alone, but to determine whether it enhances protection beyond that achieved by a single dose of A(H5) vaccine. A high-dose lethal challenge was used to avoid a ceiling effect in the A(H5)-vaccinated group. In the ferret experiment, for example, despite challenge with a high dose of 10^6^ PFU (>1,000 LD_50_) of an antigenically drifted A(H5N1) virus, a single dose of the A(H5) vaccine already provided substantial protection, with a mean maximum body weight loss of 8% and no mortality (Figure 1D and Supplementary Figure 4). A lower challenge dose could therefore result in near-complete protection after A(H5) vaccination alone, limiting the ability to detect any added benefit of seasonal vaccine priming. Even under stringent lethal challenge conditions, however, seasonal influenza vaccine priming had limited impact on A(H5) vaccine–mediated protection against A(H5N1), suggesting that heterosubtypic immune responses induced by seasonal vaccination did not translate into a clear added protective benefit compared with A(H5) vaccination alone.

A limitation of this study is that animal models do not reproduce the complex influenza immune histories of humans, who commonly experience repeated infections and vaccinations. Such immune histories may alter the magnitude, specificity, and recall of cross-reactive B-cell and T-cell responses and could modify the effect of seasonal influenza vaccine priming before A(H5) vaccination. To partially model prior seasonal influenza immunity, several experimental groups received two doses of seasonal influenza vaccines before A(H5) vaccination, with the first dose intended to establish pre-existing immunity and the second dose serving as the immediate prime before A(H5) vaccination. Even with this regimen, we observed no clear added benefit compared with A(H5) vaccination alone. Nevertheless, these animal data should not be interpreted as excluding an effect of the more complex pre-existing influenza immunity found in humans, who may have experienced repeated infections and vaccinations involving multiple influenza strains over many years. Such immune histories are impractical to reproduce in animal models.

Accordingly, clinical studies are needed to further evaluate the strategy of administering seasonal influenza vaccine prior to A(H5) vaccination. However, such studies would likely rely on immunological endpoints rather than direct assessment of protection. Evidence of cross-reactivity alone should therefore be interpreted cautiously when informing decision-making, as it may not reliably predict protection against severe or lethal A(H5N1) infection. In the absence of a clear added benefit, prioritizing seasonal influenza vaccination as a priming strategy to enhance protection against A(H5N1) during the early phase of a pandemic may offer limited value, particularly when resources may need to be directed toward A(H5)-specific vaccination and other countermeasures. Continued efforts to improve the development, availability, and deployment of antigenically similar A(H5) vaccines should remain a priority for pandemic preparedness [13,14].

## Supplementary Material

Supplemental Material

## Data Availability

All data generated or analysed during this study are included within the manuscript or supplemental data file.
