# Supplementary material for: Limited added benefit of seasonal influenza vaccination before A(H5) vaccination in mice and ferrets challenged with A(H5N1)
Source: Emerg Microbes Infect. 2026 Sep 21;15(1):2731495. doi: 10.1080/22221751.2026.2731495 (PMC13600297; doi:10.1080/22221751.2026.2731495)
Supplement: Supplementary file 1 [file TEMI_A_2731495_SM9398.docx]

**Supplemental Data**

**Limited added benefit of seasonal influenza vaccination before A(H5) vaccination in mice and ferrets challenged with A(H5N1)**

**Authors**

Masato Hatta^a*^, Ying Huang^a*^, Jeremy A. Duke^a*^, Joseph R. Rouse^a*#^, Chenchen Feng^a^, Xudong Lin^a^, Li Wang^a^, Melvyn R Almanzar-Jordan^b^, Zhu-Nan Li^a^, James Stevens^a^, Min Z. Levine^a^, David E. Wentworth^a#^, Vivien G. Dugan^a^, C. Todd Davis^a^ & Bin Zhou^a^

**Affiliation**

^a^Influenza Division, National Center for Immunization and Respiratory Diseases, Centers for Disease Control and Prevention, Atlanta, GA, USA

^b^Division of Core Laboratory Services and Response, Office of Laboratory Systems and Response, Centers for Disease Control and Prevention, Atlanta, Georgia, USA

^*^These authors contributed equally to this work.

^#^Present address: Emory University, Atlanta, GA, USA (J.R. Rouse); GSK, Cambridge, MA, USA (D.E. Wentworth)

**Table of Contents**

**Supplementary Materials and Methods	p. 2-9**

**Supplementary Table	p. 10-11**

**Supplementary Figures	p. 12-21**

**Supplementary References	p. 22**

**Supplementary Materials and Methods**

**Ethics.** All animal experiments were approved by the CDC’s Institutional Animal Care and Use Committee (IACUC) and were conducted in an Association for Assessment and Accreditation of Laboratory Animal Care International-accredited Animal Biosafety Level 3 (ABSL-3) animal facility with enhancements as required by the U.S. Department of Agriculture*^1^*.

**Biosafety and animal welfare.** All research involving HPAI A(H5N1) viruses was performed in CDC Biosafety Level 3 enhanced (BSL-3E) or ABSL-3 facilities. All personnel engaged in virus-related activities completed extensive training in relevant safety protocols and procedures, and competency to perform tasks in BSL-3E/ABSL-3 laboratories was assessed. Animal use was approved by the CDC IACUC. Animal numbers were minimised in accordance with IACUC guidelines, and procedures were implemented to reduce pain and distress. A clinical scoring system was used to determine humane euthanasia endpoints. For mice, the following criteria were applied: 1 point each—hunched posture or ruffled coat; lethargy; 15–25% loss of baseline body weight. 3 points each—≥25% loss of baseline body weight; neurological signs; unresponsiveness. Mice reaching a cumulative clinical score of ≥3 were humanely euthanised. For ferrets, the following criteria were applied: 2 points each—hunched posture or huddling; ruffled coat, piloerection, or hair loss. 3 points each—dehydration; fluctuation in body temperature. 5 points each—abnormal breathing (dyspnoea, tachypnoea, rales, or audible breaths); anaemia. 10 points each—paralysis, torticollis, moribund behaviour, or unresponsiveness; ≥20% loss of baseline body weight; reduced body temperature lasting ≥3 days; frank haemorrhage or bleeding; unrelated trauma with risk of death due to continued exposure to cage mates; or other immediate veterinary concerns. Ferrets reaching a cumulative clinical score of ≥10 were humanely euthanised.

**Cells and viruses**. Madin-Darby canine kidney (MDCK) cells (ATCC, CCL-34) were maintained in Minimum Essential Medium (MEM) supplemented with 5% fetal bovine serum at 37°C with 5% CO_2_. Virus stocks of A/Chile/25945/2023 [A(H5N1), clade 2.3.4.4b, Chile/23], A/Texas/37/2024 [A(H5N1), clade 2.3.4.4b, TX37/24], A/Washington/23/2020 [A(H1N1), WA/20], and the candidate vaccine virus (CVV) of A/American wigeon/South Carolina/22-000345-001/2021[A(H5N1), AW/SC/21] were propagated in MDCK cells and titrated by 50% tissue culture infectious dose (TCID_50_) assay using standard procedures^2,3^. rTX37-HA-P136S/A156T [A/Missouri/121/2024-like A(H5N1) virus, clade 2.3.4.4b; MO121] was generated by reverse genetics and amplified in MDCK cells^4^. All virus stocks were sequence-verified to confirm the absence of unwanted mutations and tested for exclusivity to rule out the presence of other influenza virus subtypes.

**Vaccines.** Commercially available seasonal influenza vaccines—Fluzone quadrivalent 2022–2023 formula (A/Victoria/2570/2019 [A(H1N1)], A/Darwin/9/2021 [A(H3N2)], B/Austria/1359417/2021, B/Phuket/3073/2013), Flublok quadrivalent 2022–2023 formula (A/Wisconsin/588/2019 [A(H1N1)], A/Darwin/6/2021 [A(H3N2)], B/Austria/1359417/2021, and B/Phuket/3073/2013), and Flublok quadrivalent 2023–2024 formula (A/West Virginia/30/2022 [A(H1N1)], A/Darwin/6/2021 [A(H3N2)], B/Austria/1359417/2021, and B/Phuket/3073/2013), and Flucelvax quadrivalent 2024–2025 formula (A/Wisconsin/67/2022 [A(H1N1)], A/Massachusetts/18/2022 [A(H3N2)], B/Austria/1359417/2021, and B/Phuket/3073/2013) were obtained from unused clinical supplies at the end of influenza season or directly from vaccine manufacturers. Recombinant influenza A(H5) HA protein (rH5) from AW/SC/21, with the polybasic cleavage site mutated from “REKRRKR” to “RETR” and fused to the C-terminal domain of bacteriophage T4 fibritin (foldon) and a C-terminal polyhistidine tag, was generated as described previously (Feng et al, under journal review). Recombinant influenza A(H1) haemagglutinin (HA) protein (rH1) from A/Wisconsin/588/2019 virus was purchased from Sino Biological. rH1 and rH5 were diluted in PBS and mixed with AddaS03 (InvivoGen; AS03-equivalent oil-in-water emulsion) prior to immunization.

**Mouse vaccination.** Six- to eight-week-old female BALB/c mice were used. Animals were housed in individual ventilated cages (Tecniplast USA) within a Duo-Flow unit (Lab Products, LLC) throughout all experiments. Mice were vaccinated intramuscularly with 3 µg of each HA component in Fluzone, Flublok, or Flucelvax, or with 3 µg of rH5, adjuvanted where indicated. Vaccination regimens are illustrated in Supplementary Figure 1.

Experiment 1 (Exp 1). Mice were randomised into nine groups (n = 10 per group) and vaccinated intramuscularly with rH5 derived from AW/SC/21, Fluzone quadrivalent 2022–2023 formula, or Flublok quadrivalent 2022–2023 formula. Vaccines were administered once (1x), twice (2x), or three times (3x) at 3–4-week intervals. Flublok and rH5 were adjuvanted with AddaS03, and control animals received AddaS03 alone. Serum samples were collected nine weeks after the first vaccination (two weeks after the final dose). HI titres were measured using AW/SC/21 CVV with 1% horse red blood cells (HRBC). HA-binding antibody titres in pooled sera were assessed by multiplex influenza antibody detection assay (MIADA) and Enzyme-linked immunosorbent assay (ELISA).

Experiment 2 (Exp 2). Mice were randomised into four groups (n = 15 per group) and vaccinated intramuscularly 1x or 2x with rH5 derived from AW/SC/21 or Flublok quadrivalent 2023-2024 formula. Both Flublok and rH5 were administered with AddaS03.

Experiment 3 (Exp 3). Mice (n = 15 per group) were vaccinated intramuscularly with Flucelvax quadrivalent 2024-2025 formula (without adjuvant) and/or rH5 derived from AW/SC/21 formulated with AddaS03.

**Haemagglutination inhibition (HI) assay.** HI assays were performed using 0.5% turkey red blood cells (RBC), 0.75% guinea pig RBC, or 1% horse RBC. Sera were treated with receptor-destroying enzyme (RDE; Denka Seiken Co., Ltd), heat-inactivated, and adsorbed with RBCs. Samples were then serially diluted two-fold in PBS, mixed with 8 HA units of virus, incubated at room temperature, and mixed with RBCs. HI titres are reported as the highest reciprocal dilution preventing haemagglutination.

**Enzyme-linked immunosorbent assay (ELISA).** Microtiter plates were coated with recombinant proteins overnight at 4 °C. After blocking, sera or cell culture supernatants were serially diluted, added, and incubated overnight at 4°C. Bound antibodies were detected using conjugated secondary antibodies and developed with TMB substrate. OD_450/750_ values were measured and EC_50_ values were calculated.

**Luminex assay.** Luminex**-**based multiplex influenza antibody detection assay (MIADA) was performed as described previously^5^. HA ectodomains (Ec), globular heads (G), stalk regions, and nucleoproteins (Supplementary Table 1) were coupled to magnetic COOH beads and incubated with 1:500-diluted sera. Bound IgG was detected using PE-conjugated goat anti-mouse IgG F(ab')_2_.

**Mouse challenge experiments.** Vaccinated mice were inoculated intranasally with 10^2^ TCID_50_ of Chile/23 (Exp 1) or 10^3^ TCID_50_ of TX37/24 (Exp 2 and Exp 3). Body weight and clinical signs were monitored daily for 14 days. Survival and body weight analyses excluded animals sampled for viral titration (7 mice per group in Exp 1 and 10 mice per group in Exp 2 and Exp 3). Lung samples were collected at day 3 post-infection (dpi) from 3 mice per group in Exp 1 and 5 mice per group in Exp 2 and Exp 3. Lung homogenates were titrated by TCID_50_ assay on MDCK cells as described previously^6^.

**Ferret vaccination and challenge experiment.** Male ferrets (Triple F Farms), aged 7–12 months and confirmed seronegative for circulating seasonal influenza A and B viruses, were used. Ferrets (n = 4 per group) were vaccinated intramuscularly with 15 µg of rH5 derived from AW/SC/21 or 7.5 µg of Flucelvax quadrivalent 2024–2025 formula (Supplementary Figure 3). Serum samples were collected from the cranial vena cava under anaesthesia (ketamine/xylazine) at the time points indicated in Supplementary Figure 3. Prior to the vaccination–challenge study, naïve ferrets (n = 2 per dose group) were intranasally inoculated with 10^2^ or 10^3^ TCID_50_ of MO121 to assess the virulence of the challenge virus in ferrets (Supplementary Figure 4). At week 18 after the first vaccination, ferrets were anaesthetised and inoculated intranasally with 10^6^ PFU of MO121. Survival, body weight, and body temperature were monitored daily for 13 dpi. Nasal wash samples were collected on 2, 4, 6, and 8 dpi. Organ tissues were collected from animals that reached humane endpoints. Serum samples were collected at weeks 2, 10.5, 12.5, 14, 15.5, and 17 after the first vaccination. Nasal wash and organ samples were titrated by TCID_50_ assay on MDCK cells.

**Human CD4^+^ T-cell assay.** Human peripheral blood mononuclear cells (PBMCs) were obtained from healthy adult donors (ATCC). Cytokine-derived dendritic cells were generated by isolating CD14^+^ cells (STEMCELL Technologies) and culturing them with GM-CSF and IL-4 in X-VIVO 15 (Lonza) in 12-well plates at 1.25 x 10^6^ cells per well. After six days, dendritic cells were harvested and co-cultured with donor-matched CD4^+^ T cells (isolated by magnetic separation) at a 1:20 DC:CD4 ratio in 1 mL (total 1.05 x 10^6^ cells per well). Co-cultures were left untreated or stimulated with peptide pools spanning the HA region of WI/588 [A(H1N1)] and A/Aves/Guanajuato/2023 [A(H5N1)] or human actin (JPT Peptide Technologies) and cultured for 14 days at 37 °C, 5% CO_2_. On day 14, lymphocytes were harvested and co-cultured with fresh donor-matched dendritic cells, re-stimulated with peptides, and treated with brefeldin A. After six hours, cells were processed for flow cytometry. Cells were stained using BD Horizon™ reagents (Fixable Viability Stain 780; CD4-BUV737 [RPAT4]; CD45RA-BV786 [HI100]; CD45RO-BB515 [UCHL1]; CD154-BUV563 [TRP1]; and IFN-γ-BUV805 [45.B3]) and analysed on a BD FACSymphony™ A5 using FACSDiva™, with downstream analysis in FlowJo v10.10.0.

**Human B-cell assay.** PBMCs were obtained from healthy adult donors (n = 10) (Charles River Laboratories). Cytokine-derived dendritic cells were generated as described above. After six days, dendritic cells were co-cultured with donor-matched CD4^+^ T cells at a 1:20 DC:CD4^+^ ratio (total 1.05 x 10^6^ cells per well) and left untreated or stimulated with 5 µg of an HA peptide pool spanning WI/588 [A(H1N1)] (JPT Peptide Technologies) or recombinant WI/588 HA trimer protein (Sino Biological). Cultures were incubated at 37 °C with 5% CO_2_. After three days, donor-matched B-cells were isolated by negative selection (STEMCELL Technologies) and added to the DC–CD4⁺ T-cell cultures at a 1:1 ratio (approximately 2.1 x 10^6^ cells per well). Cultures were restimulated under the same conditions and incubated for an additional 10 days. Activated memory B cells (CD19⁺ CD27⁺ CD86⁺) were quantified by flow cytometry from DC–CD4⁺ T-cell–B-cell co-cultures.

**Primary murine CD4^+^ T-cell assay.** Six-week-old female BALB/c mice were immunised twice (2-week interval) with 3 µg of adjuvanted rH1 or rH5 or left unvaccinated (naïve). Seven days post-boost, spleens were collected and processed to single-cell suspensions for *in vitro* stimulation or ELISpot. For flow cytometry, cells were plated at 1x10^6^ cells/mL in 24-well plates, (five biological replicates per condition) and stimulated overnight (18–20 hours) with 4 µg of rH1 trimer or rH5, or with PepMix^TM^ Influenza A H5 peptide pool (HA/H5N1/Guanajuato/2023) or A(H1) peptide pool (HA/H1N1/Wisconsin/2019) (JPT Peptide Technologies), or left untreated. Cells were stained with fluorochrome-conjugated antibodies against CD3, CD4, CD8 (BioLegend), and IFNγ (Thermo Fisher Scientific) and analysed on a flow cytometer (Celesta; Beckman Coulter) using FlowJo v10.10.0. For ELISpot, stimulated cells were plated at 2.5x10^5^ cells/200 µL per well using the ELISpot Plus: Mouse IFNγ kit (Mabtech) according to the manufacturer’s instructions and analysed using a Cytation 7 plate reader with Gen5 v13.1. Graphs and statistical analyses were generated using GraphPad Prism 10.

**Statistical analysis**. HI titres and organ tissue titres were analysed by one-way ANOVA with Tukey’s multiple comparisons test on log-transformed titres. To compare weight loss, the area under the curve (AUC) of body-weight loss was calculated over days 0–14 (mouse exp) and 0–13 (ferret exp) post-infection. Body-weight loss was defined as 100 minus the percentage of initial body weight recorded for each animal. For each animal, AUC was computed using the trapezoidal rule across the specified analysis window. Where measurements were missing within this interval, values were linearly interpolated, and the last observed value was carried forward to the end of the respective analysis window to standardize comparisons across animals. Group differences in AUC were assessed by one-way ANOVA, and specific comparisons between the rH5 1x group and rH5 1x groups with prior seasonal vaccination were evaluated using Tukey’s multiple comparisons test. Ferret nasal wash titres were analysed by two-way ANOVA with Tukey’s multiple comparisons test on log-transformed titres. Human and mouse CD4^+^ T-cell IFN-γ and IgG levels in human B-cell culture supernatants were analysed by one-way analysis of variance (ANOVA) with Dunnett’s multiple comparisons test. Statistical significance was defined as *p < 0.05; **p < 0.01; ***p < 0.001; ****p < 0.0001.

**Supplementary Tables**

| **Supplementary Table 1. Antigens used in Luminex-based multiplex influenza antibody detection assay (MIADA).** | | |
| --- | --- | --- |
| **Antigen** | **Domain/Protein** | **Virus strain** |
| H1 ID/18 GH | Globular head HA1 | A/Idaho/07/2018 (H1N1)pdm09 |
| H1 WI/19 GH | Globular head HA1 | A/Wisconsin/588/2019 (H1N1)pdm09 |
| H1 Syd/21 GH | Globular head HA1 | A/Sydney/5/2021 (H1N1)pdm09 |
| H1 WI/22 GH | Globular head HA1 | A/Wisconsin/67/2022 (H1N1)pdm09 |
| H3 Cam/20 GH | Globular head HA1 | A/Cambodia/e0826360/2020 (H3N2) |
| H3 Dar/21 GH | Globular head HA1 | A/Darwin/9/2021 (H3N2)-like virus |
| H3 MA/22 GH | Globular head HA1 | A/Massachusetts/18/2022 (H3N2) |
| H5.g/24 Ec | Ectodomain | A/Texas/37/2024 (H5N1) |
| H5.AW/SC/21 GH | Globular head HA1 | A/American wigeon/South Carolina/22-000345-001/2021 (H5N1) |
| H5.TX/24 GH | Globular head HA1 | A/Texas/37/2024 (H5N1) |
| B-V-WA/19 GH | Globular head HA1 | B/Washington/02/2019 (B/Victoria/2/87 lineage) |
| B-V-Aus/21 GH | Globular head HA1 | B/Austria/1359417/2021 (B/Victoria lineage) |
| B-Y-Phu/13 GH | Globular head HA1 | B/Phuket/3073/2013 (B/Yamagata/16/88 lineage) |
| H1.MI/15 Stalk | HA Stalk | A/Michigan/45/2015 (H1N1)pdm09 |
| H3.Sin/16 Stalk | HA Stalk | A/Singapore/INFIMH-16–0019/2016 (H3N2) |
| H3N2 -NP BR/10/07 | Nucleoprotein | A/Brisbane/10/2007 (H3N2) |

**Supplementary Table 2. Organ titres in infected ferrets euthanised due to disease progression.**

| **Group** | **Vaccine** | **Ferret ID  (euthanised on)** | **Cause of euthanisation** | **Organ titre logTCID_50_/g** | | | | | | | |
| --- | --- | --- | --- | --- | --- | --- | --- | --- | --- | --- | --- |
|  |  |  |  | **Lung** | **Olfactory bulb** | **Brain** | **Nasal turbinates** | **Trachea** | **Liver** | **Small intestine** | **Large intestine** |
| 1 | Flucelvax 2x + rH5 | F24-T7968 (Day 7) | Neurological signs | - | - | - | - | - | - | - | - |
| 3 | Flucelvax 1x | F24-T7976 (Day 8) | Neurological signs | - | 3.3 | 4.0 | 6.1 | 4.5 | 6.4 | 5.0 | - |
|  |  | F24-T5644 (Day 6) | Neurological signs | - | 5.4 | 3.6 | 6.6 | 5.8 | - | - | - |
|  |  | F24-T5903 (Day 6) | Neurological signs | 4.4 | 5.0 | 3.8 | 6.0 | 4.2 | - | - | - |
| 4 | rH5 2x | F24-T7942 (Day 6) | Neurological signs | - | - | - | - | - | - | - | - |

**Supplementary Figure 1. Study design of mouse experiments.**

**(A)** Experiment 1. BALB/c mice (n = 10 per group) were vaccinated intramuscularly with the Fluzone quadrivalent 2022-2023 formula, the Flublok quadrivalent 2022-2023 formula, or recombinant rH5 of A/American wigeon/South Carolina/22-000345-001/2021 virus [A(H5N1), clade 2.3.4.4b; AW/SC/21]. At week 10, mice were challenged intranasally with 10^2^ TCID_50_ of clade 2.3.4.4b HPAI A(H5N1) A/Chile/25945/2023 (Chile/23). Survival and body weight were monitored daily for 14 days post-infection (dpi). Lung samples were collected from three mice per group at 3 dpi for virus titration.

**(B)** Experiment 2. Mice (n = 15 per group) were vaccinated intramuscularly with Flublok quadrivalent 2023-2024 formula or rH5 derived from AW/SC/21. At week ten, mice were challenged intranasally with 10^3^ TCID_50_ of A/Texas/37/2024 [A(H5N1), clade 2.3.4.4b, TX37/24]. Survival and body weight were monitored daily for 14 dpi. Lung samples were collected from five mice per group on three dpi for virus titration.

**(C)** Experiment 3. Mice (n = 15 per group) were vaccinated intramuscularly with Flucelvax quadrivalent 2024-2025 formula or rH5 of AW/SC/21. At week 15, mice were challenged intranasally with 10^3^ TCID_50_ of TX37/24. Survival and body weight were monitored daily for 14 dpi. Lung samples were collected from five mice per group on three dpi for virus titration.

**Supplementary Figure 2.** **Serum titres in vaccinated mice. Seasonal vaccination alone did not induce A(H5)-specific antibody responses.**

**(A)** Luminex-based multiplex influenza antibody detection assay (MIADA) analysis of HA-binding antibodies using pooled group sera collected at week nine in Exp 1. HA proteins used in the assay are listed in Supplementary Table 1. GH, globular head; Ec, ectodomain. **(B)** ELISA analysis of HA-binding antibodies using HA proteins derived from WI588 (left panel) and A/dairy cow/Texas/24 [A(H5N1), DC/TX] (right panel) and pooled group sera collected at week nine in Exp 1. MIADA and ELISA confirmed that seasonal vaccines induced antibodies reactive with A(H1), A(H3), and influenza B HA proteins. (**C**) HI titres measured using AW/SC/21 CVV with sera collected nine weeks after the first vaccination in Experiment 1 (Exp 1), using 1% horse red blood cells (HRBC). Titres are shown as geometric means with geometric SD. The bottom dashed line indicates the assay limit of detection (10 HI), and the top dashed line indicates 40 HI. ****P < 0.0001. Sera from mice receiving seasonal vaccines alone exhibited HI titres ≤10 against A(H5), indicating the absence of A(H5)-specific antibody responses. Mice vaccinated twice with adjuvanted rH5 generated significantly higher A(H5)-specific HI titres than other groups. Although some mice in other groups exhibited low HI titres (10–20), these values remained below an HI titre of 40, a threshold commonly used as a correlate of protection in humans..

**Supplementary Figure 3. Study design of ferret challenge experiment.**

Ferrets (n = 4 per group) were vaccinated intramuscularly with Flucelvax quadrivalent 2024-2025 formula or rH5 of AW/SC/21. At week 18, ferrets were challenged intranasally with 10^6^ PFU of MO121 virus. Survival, body weight, and body temperature were monitored daily for 13 dpi. Nasal wash samples were collected on days 2, 4, 6, and 8 dpi. Organ tissues were collected from the animals that reached humane euthanasia endpoints. Serum samples were collected at weeks 2, 10.5, 12.5, 14, 15.5, and 17 after the first vaccination.

**Supplementary Figure 4. Ferret survival curve following infection with MO121.**

Naïve ferrets (n = 2 per dose group) were intranasally infected with 10^2^ or 10^3^ TCID_50_ of MO121 to assess virulence in ferrets. All animals succumbed to infection within 5 days post-infection. These findings demonstrate that MO121 is highly virulent in ferrets, with complete mortality observed even at the lower infection dose of 10^2^ TCID_50_.

**Supplementary Figure 5. Body temperature in ferrets following HPAI A(H5N1) challenge.**

Body temperature was monitored daily for 13 days following intranasal challenge with MO121 (n = 4 per group). As three ferrets vaccinated with Flucelvax 1x (Group 5) reached humane endpoints by day 8, body temperature data for the remaining ferret are shown thereafter (green arrow). Body temperature trajectories were broadly similar across groups.

**Supplementary Figure 6. Serum titres in vaccinated ferrets. Seasonal vaccination alone did not generate detectable A(H5)-specific HI antibodies.**

HI titres were measured using A/Georgia/12/2022 [A(H1N1)] and B/Connecticut/01/2021 with 0.5% turkey red blood cells (RBC); A/District of Columbia/27/2023 [A(H3N2)] with 0.75% guinea pig RBC; and AW/SC/21 CVV, TX37/24, and MO121 with 1% horse RBC. Titres are presented as geometric means with geometric SD. The bottom dashed line indicates the limit of detection (10 HI).

**Supplementary Figure 7. Cross-reactive immune responses to A(H5) HA**

**(A)** IFNγ responses in an in vitro dendritic cell–CD4⁺ T-cell co-culture system. Dendritic cells and CD4^+^ T cells isolated from PBMCs of healthy donors (n = 10) were co-cultured, primed, and restimulated with peptide pools spanning the HA of A/Wisconsin/588/2019 [A(H1N1), WI/588] or A/Aves/Guanajuato/2023 [clade 2.3.4.4b, A(H5N1)]. Activated and IFNγ-producing T cells were quantified by flow cytometry gated on CD4⁺ CD45RO⁺ CD154⁺ IFNγ⁺. **(B)** Human B-cell responses. Activated memory B cells (CD19⁺ CD27⁺ CD86⁺) in DC–CD4^+^ T-cell–B-cell co-cultures stimulated with either an H1 HA peptide pool or recombinant A(H1) HA protein (WI/588) (n = 10 donors) were quantified by flow cytometry. Comparisons without marked significance values were not significant by Dunnett’s multiple comparisons test.

**Supplementary Figure 8. Cross-reactive IFNγ responses to A(H1) and A(H5) HA antigens in mouse CD4⁺ T cells in vitro. Vaccination with rH1 or rH5 elicited IFNγ responses to both A(H1) and A(H5) antigens.**

**(A–B)** BALB/c mice were vaccinated with recombinant A(H1) HA protein (rH1) or recombinant A(H5) HA protein (rH5), or left unvaccinated, and boosted at week two. Splenocytes collected one week post-boost were stimulated overnight with A(H1) or A(H5) HA peptide pools or with rH1 or rH5. (A) IFNγ ELISpot counts in splenocytes (2.5 × 10⁵ cells/well). (B) Flow-cytometric quantification of IFNγ⁺ CD44⁺CD62L⁺ CD4⁺ T cells. The dotted line indicates the mean of untreated controls.

**Supplementary References**

1.	Chosewood LC, Wilson DE, Centers for Disease Control and Prevention (U.S.), National Institutes of Health (U.S.). Biosafety in microbiological and biomedical laboratories. 5th ed. Washington, D.C.: U.S. Dept. of Health and Human Services, Public Health Service, Centers for Disease Control and Prevention, National Institutes of Health; 2009.

2.	World Health Organization. WHO manual on animal influenza diagnosis and surveillance. 2002. iris.who.int/handle/10665/68026.

3.	Balish AL, Katz JM, Klimov AI. Influenza: propagation, quantification, and storage. *Curr Protoc Microbiol* 2013; **Chapter 15**: 15G 1 1-G 1 24.

4.	Wang L, Hatta M, Feng C, et al. Impact of naturally occurring hemagglutinin substitutions on antigenicity and fitness of influenza A(H5N1) virus. *Npj Viruses* 2025; **3**(1): 72.

5.	Li ZN, Liu F, Jefferson S, et al. Multiplex Detection of Antibody Landscapes to Severe Acute Respiratory Syndrome Coronavirus 2 (SARS-CoV-2)/Influenza/Common Human Coronaviruses Following Vaccination or Infection With SARS-CoV-2 and Influenza. *Clin Infect Dis* 2022; **75**(Suppl 2): S271-S84.

6.	Hatta M, Hatta Y, Choi A, et al. An influenza mRNA vaccine protects ferrets from lethal infection with highly pathogenic avian influenza A(H5N1) virus. *Sci Transl Med* 2024; **16**(778): eads1273.
